# Application of machine learning in SNP discovery

**DOI:** 10.1186/1471-2105-7-4

**Published:** 2006-01-06

**Authors:** Lakshmi K Matukumalli, John J Grefenstette, David L Hyten, Ik-Young Choi, Perry B Cregan, Curtis P Van Tassell

**Affiliations:** 1 US Department of Agriculture, ARS, Beltsville Agricultural Research Center, Bovine Functional Genomics Laboratory, Beltsville, MD 20705, USA.; 2 Bioinformatics and Computational Biology, George Mason University, Manassas, VA 20110, USA.; 3 US Department of Agriculture, ARS, Beltsville Agricultural Research Center, Soybean Genomics and Improvement Laboratory, Beltsville, MD 20705, USA.

## Abstract

**Background:**

Single nucleotide polymorphisms (SNP) constitute more than 90% of the genetic variation, and hence can account for most trait differences among individuals in a given species. Polymorphism detection software PolyBayes and PolyPhred give high false positive SNP predictions even with stringent parameter values. We developed a machine learning (ML) method to augment PolyBayes to improve its prediction accuracy. ML methods have also been successfully applied to other bioinformatics problems in predicting genes, promoters, transcription factor binding sites and protein structures.

**Results:**

The ML program C4.5 was applied to a set of features in order to build a SNP classifier from training data based on human expert decisions (True/False). The training data were 27,275 candidate SNP generated by sequencing 1973 STS (sequence tag sites) (12 Mb) in both directions from 6 diverse homozygous soybean cultivars and PolyBayes analysis. Test data of 18,390 candidate SNP were generated similarly from 1359 additional STS (8 Mb). SNP from both sets were classified by experts. After training the ML classifier, it agreed with the experts on 97.3% of test data compared with 7.8% agreement between PolyBayes and experts. The PolyBayes positive predictive values (PPV) (i.e., fraction of candidate SNP being real) were 7.8% for all predictions and 16.7% for those with 100% posterior probability of being real. Using ML improved the PPV to 84.8%, a 5- to 10-fold increase. While both ML and PolyBayes produced a similar number of true positives, the ML program generated only 249 false positives as compared to 16,955 for PolyBayes. The complexity of the soybean genome may have contributed to high false SNP predictions by PolyBayes and hence results may differ for other genomes.

**Conclusion:**

A machine learning (ML) method was developed as a supplementary feature to the polymorphism detection software for improving prediction accuracies. The results from this study indicate that a trained ML classifier can significantly reduce human intervention and in this case achieved a 5–10 fold enhanced productivity. The optimized feature set and ML framework can also be applied to all polymorphism discovery software. ML support software is written in Perl and can be easily integrated into an existing SNP discovery pipeline.

## Background

### Machine learning

Machine learning (ML) is the study and computer modeling of learning processes including the acquisition of new declarative knowledge, organization of new knowledge into general effective representations, and the discovery of new facts through observation and experimentation. Machine learning programs are advantageous in many cases where the input/output pairs can be specified, but the concise relationship between the input/output pairs is not known. Machine learning programs can help in extracting the complex relationships and correlations hidden in large data sets (a process sometimes known as data mining).

The prediction accuracy of different machine learning programs varies and depends on the type of problem, dataset and the algorithm used. Examples of application domains include protein classification[1] tissue classification for different types of cancer[2], protein secondary structure prediction [3], text mining[4], protein-protein interactions[5] and RNA binding proteins[6]. The most common ML algorithms include decision trees, production rules, support vector machines, naïve Bayes, neural networks, and genetic algorithms. There are several free software suites available, including Weka [7], C4.5 [8], and GIST [9].

### SNP discovery

Single nucleotide polymorphisms (SNP) are single base variations or short insertions/deletions in the nucleotide sequence from different individuals or between homologous sequences within an individual. SNP markers are relatively dense and abundant when compared to other marker types. SNP can be used for distinguishing between individuals and species, genetic analysis of disease and complex traits, assessment of linkage disequilibrium (LD), haplotype map generation, pharmacogenomics, etc. In a large scale SNP discovery project after sequencing and assembly of the sequences from different individuals/genotypes, candidate SNP are usually identified by using programs like PolyBayes [10] or PolyPhred [11].

PolyPhred is more commonly used for SNP detection in re-sequencing data as it can detect the heterozygotes. PolyBayes was designed to statistically detect SNPs in multiple alignments of overlapping EST or shotgun sequences. However, PolyBayes is more suitable for soybean re-sequencing data as soybean is an extensively in-bred species and most "heterozygous" bases observed would be due to single base differences between paralogs. Each of the candidate SNP identified by PolyBayes is expertly verified by visual inspection. The criteria for a good SNP include high quality phred scores of the varying base position, minor allele frequency, agreement between the forward and reverse reads and co-variance of polymorphisms for the same genotype. This paper addresses the issue of reducing the amount of intervention required by human experts.

### Application of machine learning in polymorphism discovery

**Figure 1:**
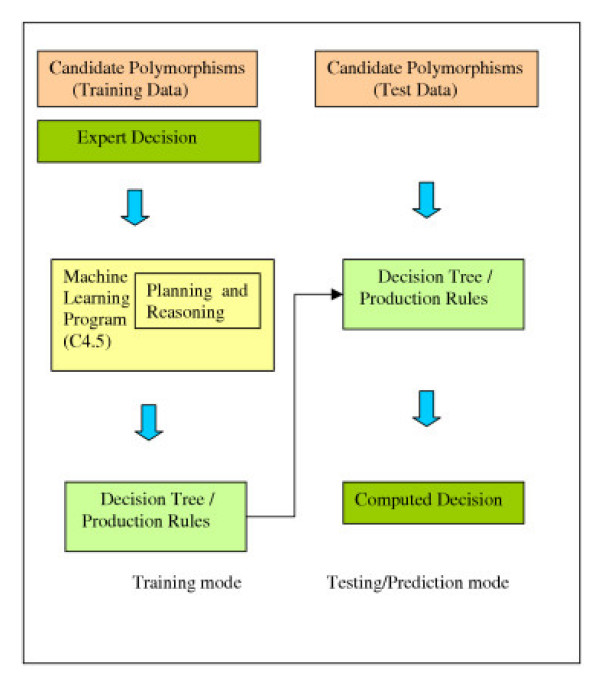
**Application of machine learning program in training and test/prediction modes**. The left side of the flow chart represents the training mode where the input features along with the expected output are fed simultaneously to the ML program. The program then analyzes the data and generates a model in the form of decision tree or a set of production rules. The right side of the flow chart represents the testing or prediction mode where the model generated in the training mode is used to evaluate a new set of input features for predicting an expected output.

To reduce the cost of expert intervention in polymorphism discovery, we applied the ML program C4.5 [8] to train a SNP classifier model from an expert reviewed dataset. The classifier can be subsequently used to predict unseen cases. C4.5 was chosen because it gives prediction for a previously unseen case and also generates a decision tree (or a set of production rules) that can be interpreted to understand the expert evaluation process in more detail (Fig. 1). C4.5 program is freely available with open source C code that can be compiled and executed on nearly any platform. A decision tree consists of a number of nodes, where each node corresponds to a test based on a single feature. At each point in the construction of the decision tree, C4.5 selects the feature to test based on maximum information gain. The goal is to generate a minimum size tree that correctly classifies all the elements in the training set. The size of the tree is the number of nodes (decision nodes + leaves) and the numbers of errors are the misclassified cases. The program also gives projected prediction accuracy for unseen cases. Production rules are generated by starting with an initial rule set and iteratively improving the rule set using heuristic techniques or by first generating a tree and then converting the tree into an equivalent rule set and finally pruning unnecessary rules.

Specific objectives of this study were to

(1) identify features that can influence the polymorphism scoring decisions,

(2) develop a software program for applying the ML program C4.5 to SNP features,

(3) optimize features to improve prediction accuracy of the classifier,

(4) use optimized feature set on a large dataset for improved prediction accuracy.

## Results

### Training and test data

The training/test candidate polymorphism data for implementing ML algorithms was extracted from a large-scale soybean STS amplification and sequencing project. For the primers designed the STS that produced a single discrete band PCR product on agarose gel electrophoresis were sequenced. A total of 3332 STS comprising 20 Mb were sequenced from both directions in 6 inbred individuals representing each of 6 diverse soybean genotypes previously identified by Zhu et al [12]. Most of these data have a uniform sequence depth of 6 reads in each direction. These data were split into a training set consisting of 1973 STS (12 Mb sequence) with 27,275 candidate polymorphisms (identified by the PolyBayes program) and a test set of 1359 STS (8 Mb sequence) with 18,390 candidate polymorphisms. Subject matter experts classified the above candidate polymorphisms as 2969 true and 24,306 false in the training set and 1435 true and 16,955 false in the test set.

### Application of PolyPhred

PolyPhred is a commonly used tool for polymorphism identification in re-sequencing data as it can detect heterozygotes. Application of PolyPhred on the test data resulted in only 1346 candidates (743 true positive, 563 false positive). Thus the sensitivity of this tool for this dataset is only 54.5% with a positive predictive value of 58.1%. The poor performance of polyphred in this case may be partly because of the un-suitability of this tool for in-bred species like soybean where heterozygosity is mostly due to sequencing noise or co-amplification of paralog sequences. In the latter case all genotypes appear to be "heterozygous" at a given position and PolyPhred identifies a SNP at that position.

### Feature selection and optimization

While ML programs are useful for creating classifiers based on a given set of features, the selection of the relevant features is often a challenging task, usually requiring an iterative approach. We first selected a set of 10 features that were likely to influence the human expert when classifying a putative SNP. These features were then optimized by modifying the existing features and adding new features that helped in improving the prediction accuracy. The final set of optimized features is given in Table 1.

**Table 1:** Final set of optimized features chosen for machine learning

**Feature Number**	**Feature**	**Variable Type**
1	Sequence depth	Continuous
2	Variation type	transition transversion indel
3	PolyBayes probability	Continuous
4	Frequency of major allele	Continuous
5	Frequency of minor allele	Continuous
6	Relative distance from closest end	Continuous
7	Agreement in the forward and reverse reads	Continuous
8	Maximum quality of the major allele	Continuous
9	Maximum quality of the minor allele	Continuous
10	Average quality of major allele	Continuous
11	Average quality of minor allele	Continuous
12	Haplotype of second variation	Continuous
13	Local average quality	Continuous
14	Overall average quality	Continuous
15	Alignment quality	Continuous
16	Common repeats	Repeat_type

A detailed definition and explanation of these features is given the methods section. The values for the features can be continuous in a given numerical range or discrete with limited options.

Description of these features is given in the methods section. The optimization runs for feature selection are discussed in more detail at the website containing supplementary material [21].

### Application of C4.5 on training data and evaluation on test dataset

A software program was developed to extract the features described above, execute C4.5 and analyze the results. The software features are described in the methods section 5.2. A five fold cross-validation was performed on the training data of 27,275 cases using both available options with C4.5 i.e., decision tree and production rules. To perform the cross-validation, the data were divided into five parts and the ML classifier was recursively trained on four parts and tested on the remaining part (analogous to a jack-knife procedure). The performance of the resulting decision trees/production rules was evaluated (definitions of the measures used are given in the methods section 5.3). The average prediction accuracies of validation runs were above 96.5%.

A new decision tree and production rule set was constructed using the training set of 27,275 cases and was then applied to the test set of 18,390 previously unseen cases. Results are shown in Table 2.

**Table 2:** Comparison of ML and PolyBayes on test data set

**Measure**	**Decision Tree**	**Production Rules**	**PolyBayes**
**TP**	1153	1202	1435
**TN**	16,748	16,706	NA
**FP**	207	249	16,955
**FN**	282	233	NA
**Accuracy**	97.3	97.4	7.8
**Sensitivity**	80.3	83.8	100 (Set)
**Specificity**	98.7	98.5	NA
**Positive Predictive Value**	84.8	82.8	7.8
**Negative Predictive Value**	98.3	98.6	NA

We define the following terms used to contrast ML performance with PolyBayes: We say that a SNP prediction program produces a true positive (TP) if it predicts a SNP that is judged true by the expert. Likewise, a false positive (FP) is a predicted SNP that is judged false by the expert, a true negative (TN) is a prediction of a non-SNP that concurs with the expert, and a false negative (FN) is a failure to identify a SNP that is identified by the expert. Also the following parameters were used to measure the performance of the ML output: Accuracy (i.e., fraction of candidate SNP correctly classified), sensitivity (i.e., fraction of positive outcomes correctly identified), specificity (i.e., fraction of the negative outcomes correctly identified), positive predictive value (i.e., fraction of predicted SNP being true) and negative predictive value (i.e., fraction of predicted false SNP being correctly classified)

Accuracy = (TP + TN)/total

Sensitivity = TP/(TP + FN)

Specificity = TN/(FP + TN)

Positive Predictive Value (PPV) = TP/(TP + FP)Negative Predictive Value (NPV) = TN/(TN + FN)

Application of machine learning program substantially reduces the number of false positives from 16,955 to only about 250. Other statistical measures also demonstrate considerable advantage in the application of machine learning.

Since only PolyBayes predictions were used in both training and test data sets, the true negative and false negative terms for PolyBayes are not known for this study. The prediction accuracy of ML algorithms using both decision trees and production rules was above 96%. Also implementation of ML algorithms resulted in 10 fold increase in productivity by increasing the PPV from 7.8% to 84.8%.

The number of false positives in the training/test data set is expected to decrease by increasing the PolyBayes threshold probability values. From Table 3, it can be observed that the numbers of TP and FP both increased with the PolyBayes probability score, to reach a maximum PPV of 16.7% with a PolyBayes posterior probability of 1.00. By using machine learning the overall PPV can be enhanced to 82.8%. The ML PPV did not improve with the confidence values from the ML algorithm; hence confidence values were not informative.

**Table 3:** Comparison of positive predictive values (PPV) from PolyBayes and ML predictor

	**PolyBayes**		
**Probability (P)**	**TP**	**FP**	**PPV**

**P ≤ 0.60**	20	1756	1.1
**0.60 < P ≤ 0.70**	38	1529	2.4
**0.70 < P ≤ 0.80**	31	1683	1.8
**0.80 < P ≤ 0.90**	45	2015	2.2
**0.90 < P ≤ 0.95**	50	1613	3.0
**0.95 < P ≤ 0.97**	53	1055	4.8
**0.97 < P ≤ 0.99**	148	2069	6.7
**P = 1.00**	1050	5235	16.7
**Overall**	1435	16955	**7.8**

	**ML Predictor**		

	**TP**	**FP**	**PPV**
**Overall**	1153	207	**84.8**

TP: True Positive, FP: False Positive,

Positive predictive value (PPV) = TP/(TP + FP).

The number of true positives in the dataset can be increased by using stringent PolyBayes posterior probability cut-off values. However, even when the posterior probability value is set to the maximum of 1.0 the positive predictive value with PolyBayes is less than 20%. Application of machine learning showed a 5–10 fold increase in the PPV at different PolyBayes posterior probability values.

**Figure 2:**
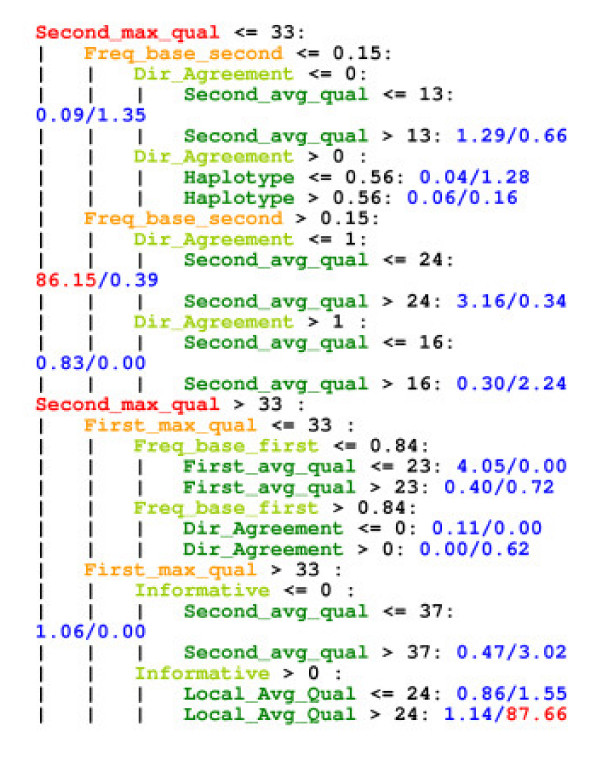
**Simplified Decision Tree**. The decision tree after pruning has 491 nodes. The figure above shows only the top four layers of nodes that indicate the most critical features in the ML decision making process. A detailed version of this tree is at the website [21].

Subject matter expert re-analysis of a small sample of 116 candidate SNP where ML algorithm prediction did not agree with the expert decision revealed that some of the decisions were subjective and those can influence the ML algorithm. Out of the 116 re-evaluated calls, 52 calls were re-classified and 64 calls were confirmed to be correctly annotated by the expert previously. Some of the reasons cited for re-classification were SNP calls made even with poor sequence quality (32), misalignment of bases (7), deletions overlooked (2) and simple sequence repeat polymorphism (SSR) (3). Similar considerations may account for some of the 19.7% SNP not flagged by the ML algorithm. Subject matter experts scored differently in some cases especially with low reliability ambiguous sequence data and others were errors due to oversight. Decision trees and production rules revealed interesting insights in the expert decision criteria and helped improve ML features (Fig. 2).

### ML programs other than C4.5

Several ML algorithms other than C4.5 such as neural networks, SVM and genetic algorithms are being widely used. In this study we explored the use of feed forward neural networks (Matlab toolbox) for the same dataset with different options (number of nodes, layers and training algorithms) and obtained similar overall accuracies (97%) and marginal increase in PPV up to 87%. Details of these runs are provided as supplementary materials on the website. C4.5 is free software that can be implemented with relative ease with an equivalent performance for the options tested with neural networks.

## Discussion

The soybean has a complex genome, with studies suggesting multiple rounds of duplication of some genomic regions resulting in high paralog frequency [13-15], This complex genome structure may account to some extent for observing a higher number of false positive SNP from the PolyBayes analysis. Soybean is an extensively inbred species and the variation between the two homologous chromosomes is negligible. Hence, PolyBayes, rather than PolyPhred, was used for SNP discovery for these data. This paper only attempts to automate the expert confirmation process. To evaluate the performance of expert scoring a different ML training and test dataset is required. Confirmed SNP are currently being mapped to the soybean genome.

PolyBayes and PolyPhred are primarily used for analyzing small sequence datasets. Large-scale, genome-wide SNP discovery projects routinely use customized versions of neighborhood quality standard (NQS) [16]. NQS is a set of rules for SNP filtering based on the sequence quality of the varying base along with the quality of the neighborhood bases.

The application of ML is not dependent on the screening method used, but instead can be used with any of the aforementioned tools that are used for SNP discovery. The ML tool simply automates the rule development and can be applied to small and large datasets where good training data are available.

Machine learning has been applied to polymorphism discovery from amplified STS and was demonstrated to have a positive impact in polymorphism discovery. The optimized ML feature set can be tailored and applied to other instances of polymorphism discovery and ML in general can be applied to other genomics and bioinformatics decision making problems.

## Conclusion

Major efforts are now being undertaken in polymorphism discovery in several species, including humans, to help characterize population differences. ML can enhance the prediction accuracies of these existing programs. In this study we have

• Identified a feature set to enhance polymorphism prediction accuracies,

• Used the ML program C4.5 to generate a decision tree (production rules) from a training set to obtain an overall prediction accuracy of 97% in the five fold cross-validation and from a new unseen test set,

• Enhanced the PPV by 5- to 10-fold compared to using only PolyBayes for these data, and

• Developed an open source software package in Perl to apply machine learning in polymorphism discovery with modules for computation of the values of the optimized feature set, execution in test mode to retrieve predictions, a graphical interface for easy SNP scoring and a provision to store feature values of new data for further improvements. The system and source code along with test and training data are provided in Additional file 1.

ML enhanced the prediction efficiency overall (97%) along with the PPV (85%) in soybean sequences with a complex genome that might have contributed to high false positives being predicted by PolyBayes. Hence the PPV with sequences from other genomes may vary.

## Methods

### Feature selection and optimization

While ML programs are useful for creating classifiers based on a given set of features, the selection of the relevant features is often a challenging task, usually requiring an iterative approach. We first selected a set of 10 features that were likely to influence the human expert when classifying a putative SNP. These features were then optimized by modifying the existing features and adding new features that helped in improving the prediction accuracy. The final set of optimized features is given in Table 1. The optimization runs for feature selection are discussed in more detail at the website containing supplementary material [21].

#### Sequence depth

Sequence depth (feature #1) is the count of number of sequences in the alignment at the position of variation. All sequences in the alignment may not overlap at the position of variation; hence this number is different from the total number of the sequences in the alignment. Having more sequence reads at the polymorphic position improves the confidence in making a judgment. We defined the sequence depth sd as:

sd = a + t + g + c + i

Where a, t, g, c and i are the number of occurrences of A, T, G, C and insertions/deletions(indel), respectively, at the position of variation

#### Variation type

Variation type (feature #2) can be a transition, a transversion or an indel. In humans, transitions are reported to be more common than transversions with a ratio of 2 to 1 [17], however in soybean [12] transitions occur at nearly equal rates as transversions (48 vs 52%). ML programs can learn from the training data and may give more weight based on the variation type observed for a given species. Also, some general rules may evolve when the polymorphism is an indel.

#### PolyBayes probability

The PolyBayes program [10] assigns a Bayesian posterior probability value (feature #3) for each called SNP using the frequency priors given for observing a variation at that position. However, the frequencies can be estimated for only very few species and can vary by region (hotspots vs. islands). The PolyBayes engine with its default values still makes a very good judgment in identifying high quality SNP from large sequence alignment data. The default prior probability of PolyBayes is in close agreement with the observed average polymorphism rate in soybean genome.

However, for each STS the localized values tend to be highly variable, which cannot be accounted for by PolyBayes. Table 3 shows an improvement in prediction accuracy by increasing the PolyBayes threshold probability values. Hence, this feature was included to improve the performance of the ML algorithm.

#### Base frequencies

The number of occurrences of different bases including indels at the position of variation is important in determining a polymorphic position. Frequencies of the first (major allele) and the second (minor allele) most commonly observed bases at the positions of variation were selected and are expressed as a ratio with respect to sequence depth. (features # 4, 5)

We first computed the sorted values:

(s1, s2, s3, s4, s5) = sort descending (a, t, g, c, i)

where a, t, g, c, and i are defined as above. We then computed the frequency ratios:

Frequency of the major allele = s1/sd

Frequency of the minor allele = s2/sd

#### Relative distance

Sequence quality at the ends of the alignment tends to be poor due to inherent limitations of current sequencing technology. Sequence alignment programs like Phrap do not trim the sequence for low quality and use the low quality sequence information to identify overlap between any two reads for creating longer alignments. Hence polymorphisms detected at either end of an alignment tend to be unreliable. To account for this factor, the polymorphism position was represented as the ratio of the distance in the consensus sequence from the closest end, or the relative distance (feature # 6)

rd = p/L (if p/L ≤ 0.5)

rd = 1 - p/L (if p/L > 0.5)

Where rd is the relative distance, p is the polymorphic position in the alignment and L is the length of the consensus sequence.

#### Directional agreement

The PCR amplified DNA fragments or sequence tagged sites (STS) were routinely sequenced in both directions. This often results in a sequence overlap. If the polymorphic site lies on the overlapping sequence segment the polymorphic base is more informative. Hence the number of such sequences having the same base when sequenced from both directions was chosen as one of the features (feature # 7).

#### Sequence quality

Sequence quality of the bases at the polymorphic site is a very important parameter when considering possible SNP. Frequently, variation is observed because of a poor quality base, and such polymorphism will be rejected in the scoring process. Since there will be several reads at the polymorphic site, aggregated features were defined:

These values were derived as follows. As described in 2.4 the sorted frequency values of bases (s1, s2, s3, s4 and s5) were calculated for the polymorphic position. Let b1 be the base for the major allele (s1) and b2 be the base for the minor allele (s2), then the aggregate parameters for all the sequencing reads in the polymorphic position were defined as maximum qualities maxQ(b1), maxQ(b2) and average qualities avgQ(b1), avgQ(b2) (features # 8,9,10 and 11).

#### Haplotype variation

**Figure 3:**
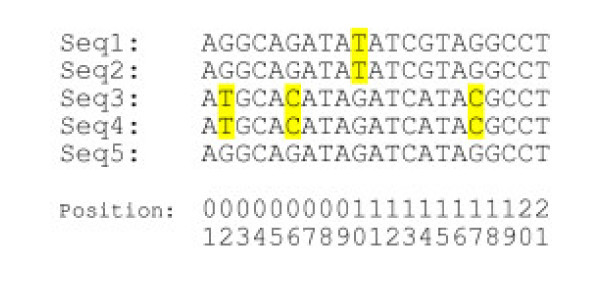
**SNP likelihood in sequences showing common variation**. The positions indicated in dark grey are the polymorphic positions. Sequences 2 and 4 show common variation at two positions in the sequence alignment, and hence these polymorphisms are more likely to be real than the common variation shown in sequences 1 and 5 or the variation in sequence 3.

In a previous report [12], our group observed that in 500–700 bp soybean STS containing two or more SNP a high level of linkage disequilibrium was present. Batley et al [18,19] observed that co-segregation of the SNP pattern between multiple SNP loci in an alignment as one of the important factors in SNP discovery. Because of the resulting haplotype structure, the SNP allele present at any one position in a fragment amplified from a particular genotype is highly predictive of other alleles in that fragment. Thus if variations are observed in the same sequence at different polymorphic sites and these polymorphisms correspond to one of the two or three haplotypes present in the STS then the polymorphisms detected in these positions are more likely to be true (Fig. 3). To capture this concept a feature called haplotype factor (feature # 12) was defined and the algorithm for the calculation is given in Table 4.

**Table 4:** Algorithm for haplotype variation factor determination

*N is the total number of polymorphic positions*
*For each polymorphic position i = 1 to N*
*List of chromatograms having the major allele b1, minor allele b2 are b1(i) and b2(i) respectively*.
*Set Sum(HapVariationFactor) to zero*.
*For each of the polymorphic position j = 1 to N and i ≠ j*
*List of chromatograms having the major allele b1 and minor allele b2 are b1(j) and b2(j)*
*c(i,j) is the number of elements (chromatograms) common in b2(i) and b2(j) and t is the number of elements in b2(j) then*
*Sum(HapVariationFactor) += c(i,j)/t*
*End of For loop*
*HaplotypeFactor = Sum(HapVariationFactor)/N*
*End of For loop*

Haplotype variation factor is defined as a measure of co-variance observed in the same chromatogram across different SNP loci. For each SNP locus the fraction of number of co-variances (observing minor alleles at different SNP locus on the same chromatogram) with respect to total number of minor alleles observed is first calculated. These values are then summed for all positions and the mean value (haplotype variation factor) is calculated by dividing by the total number of polymorphisms.

#### Alignment quality

Misalignment of bases caused by sequence alignment programs sometimes result in an erroneous SNP call. To account for that factor, alignment quality (features # 15) was incorporated. This feature is calculated as follows:

(1) Create a list of all chromatograms that had coverage in the polymorphic position.

(2) Set the alignment penalty parameter to zero.

(3) For each chromatogram from the list above

▪ In the neighborhood (+/- 5 bases) of the polymorphism site all the mismatches with the consensus sequence are given a penalty and the penalty is more if the mismatch is an indel.

▪ Ignore the mismatches at other polymorphic positions in the neighborhood.

The alignment penalty parameter is then scaled from 0 to 1, where 1 is the highest quality alignment with no mismatches or indels in the neighborhood of the polymorphic base.

#### Repeat masking

The RepeatMasker program is used to identify low complexity DNA sequences and common repeats that are specific for a given species [20]. Common repeats observed were (A)n, A-rich, AT_rich, (CAG)n, (GAA)n, (GA)n, GC_rich, (TA)n, (TATG)n and (TC)n. Some of the SNP are due to changes in the number of repeat elements also referred to as simple sequence repeats (SSR). This was provided as an optional feature to be able to distinguish SNP from SSR (feature # 16).

### Implementation of ML in polymorphism discovery

A software package to support ML in polymorphism discovery was written in Perl that uses other open source Perl modules from Bioperl [22] and CPAN [23]. The software code will be portable to most platforms where Perl can be executed. The software has modules for:

• Extracting the ML features (Table 1) from the output files generated by the sequence assembly program (phredPhrap or CAP3) and polymorphism detection programs (PolyBayes or PolyPhred) and creating a data file in the format required for C4.5 execution.

• Running the C4.5 programs to obtain predictions for test cases using either a decision tree or production rules. These programs run in a fraction of the time required to run phredPhrap.

• Creating a graphical display similar to the Consed viewer on a web page along with the ML prediction to facilitate easy scoring of polymorphisms.

• Distinguishing certain polymorphic heterozygous positions that are common in all genotypes sampled (ignored by PolyPhred). This can help in distinguishing paralogs.

This package [see Additional file 1] can also be integrated as a part of a polymorphism analysis pipeline. The software features were optimized for discovering SNP in homologous chromosomes. For enhancing the prediction accuracies of heterozygous SNP (identified by PolyPhred) additional features can be incorporated. Similarly, the feature set can be tailored for other instances of polymorphism discovery (WGS, EST) depending on the availability of sequence and quality information.

## List of abbreviations

SNP: Single nucleotide polymorphism

EST: Expressed sequence tag

SSR: Simple sequence repeat

STS: Sequence tagged site

PCR: Polymerase chain reaction

ML: Machine Learning

TP: True positive

TN: True negative

FP: False positive

FN: False negative

PPV: Positive predictive value

NPV: Negative predictive value

WGS: Whole genome shotgun sequence

CPAN: Comprehensive Perl Archive Network

NQS: Neighborhood quality standard

## Authors' contributions

LM, JG dealt with the computational aspects in the implementation of Machine learning programs. IC, DH and PC performed the sequencing, SNP data generation and suggested important features for ML implementation. CVT provided overall guidance for this project and also performed the statistical analysis. All authors read and approved the final manuscript.

## Supplementary Material

Additional File 1 contains the system and source code along with the training and test data for implementation of the machine learning algorithms in polymorphisms discovery. Click here for file
